# Determination of Matrine in Rat Plasma after Oral Administration of Novel Korean Herbal Medicine KIOM-MA128 and Application of PK

**DOI:** 10.1155/2015/431632

**Published:** 2015-02-16

**Authors:** Hyun-moon Back, Byungjeong Song, Jung-woo Chae, Hwi-yeol Yun, Jin Yeul Ma, Kwang-il Kwon

**Affiliations:** ^1^College of Pharmacy, Chungnam National University, Daejeon 305-764, Republic of Korea; ^2^Herbal Medicine Improvement Research Center, Korea Institute of Oriental Medicine, Daejeon 305-811, Republic of Korea

## Abstract

KIOM-MA128 is a novel Korean herbal medicine with antiatopic, anti-inflammatory, and antiasthmatic effects. Matrine is thought to be a potential chemical marker of KIOM-MA128, but pharmacokinetic studies on KIOM-MA128 had not been performed. This study describes a simple and rapid method using high-performance liquid chromatography-tandem mass spectrometry (HPLC-MS/MS) to determine the concentration of matrine in rats plasma after administration of KIOM-MA128. The isocratic mobile phase consisted of methanol and distilled water, and the flow rate was 0.15 mL/min. The accuracy and precision of the assay, as well as stability tests, were performed in accordance with FDA regulations for the validation of bioanalytical methods. The half-life and *T*_max_ of matrine after administration of KIOM-MA128 were 4.29 ± 2.20 h and 1.8 ± 1.23 h, respectively. *C*_max_ and AUC_inf_ of matrine after administration of KIOM-MA128 at 4 g/kg and 8 g/kg were 595.10 ± 182.91 ng/mL, 5336.77 ± 1503.84 ng/mL·h and 850.46 ± 120 ng/mL, 9583.10 ± 888.92 ng/mL·h, respectively. The validated method was successfully applied to a pharmacokinetic study in rats after oral administration of KIOM-MA128.

## 1. Introduction

Atopic dermatitis (AD) is a chronic and relapsing eczematous dermatitis accompanied by the formation of intraepidermal vesicles during the acute phase and scaly pachydermatosis during the chronic phase [1]. T cells and eosinophils are thought to play a major role in the pathogenesis of this disease [2]. Skin lesions in AD show a mononuclear cell infiltrate consisting of activated CD4^+^ T cells [3], and many studies have suggested that the development and pathogenesis of AD are associated with immunological abnormalities, such as increased immunoglobulin E (IgE) levels [4–7]. AD causes periodic hysterical pruritus, and because of this, pruritus skin lesions recur or worsen with purpuric papules, eczematous lesions, and lichenification [8]. The incidence rate of AD in infants and children is especially high, but effective treatment is not available. In general, topically applied corticosteroids have been the standard for the majority of patients with AD [9]. However, the repeated use of topical corticosteroids can have numerous adverse systemic effects [10, 11].

KIOM-MA is a novel Korean herbal medicine that consists of 18 herbs, mainly* Radix Sophorae*,* Radix Glycyrrhizae*, and* Cnidii Rhizoma*. KIOM-MA128 is the fermented form of KIOM-MA produced by* Lactobacillus acidophilus*. The anti-inflammatory effect of this herbal drug has already been reported [12], and KIOM-MA128 is also known to have antiasthmatic and antiatopic effects [13, 14]. According to previous studies [13], KIOM-MA128 decreased clinical features and IgE levels for the treatment of AD in a dose-dependent manner, and oral administration may be useful for clinical application. The effect of KIOM-MA128 has been characterized, but the pharmacokinetics of KIOM-MA128 after oral administration has not been explored.

Matrine is a type of alkaloid and a major chemical marker of* Radix Sophorae* that affects the expression of inflammatory cytokine production and can be used to treat AD [15].* Radix Sophorae* is an important formulation component, and matrine is also a potential chemical marker of KIOM-MA128 with anti-AD and anti-inflammatory effects. Therefore, this study developed simple and rapid method for determination of matrine which is one of the major compounds of KIOM-MA128 in rat plasma using high-performance liquid chromatography-tandem mass spectrometry (HPLC-MS/MS) with electrospray ionization. And pharmacokinetics of KIOM-MA128 was determined after oral administration at the dose of 4 g/kg, 8 g/kg.

## 2. Materials and Methods

### 2.1. Materials and Reagents

KIOM-MA128 was provided by the Korean Institute of Oriental Medicine (Daejeon, Korea) [12, 13]. Matrine was provided by the Korea Food & Drug Administration (KFDA, Osong, Korea). Acetaminophen, used as an internal standard (IS), was purchased from Sigma-Aldrich Canada Co. (Oakville, ON, Canada), and HPLC-grade methanol was obtained from Merck (Darmstadt, Germany). A PURELAB Ultra system from ELGA (Marlow, UK) was used in the laboratory to produce deionized water.

### 2.2. MS Conditions

To optimize MS conditions, a solution containing 100 ng/mL of matrine was infused into the tandem mass spectrometer (API 2000; Applied Biosystems, Foster City, CA, USA) at a flow rate of 10 *μ*L/min. The turbo ion spray interface was operated in positive ion mode at 5500 V for spray voltage and ion spray temperature was set to 350°C. The precursor ions of the analytes were optimized as protonated molecular ions, [M + H]^+^. Matrine was quantified using the multiple reaction monitoring mode and the peak-area ratio method with an IS.

### 2.3. Sample Preparation

To prepare calibration and quality control (QC) samples, 5 *μ*L of the matrine standard solution was added to 45 *μ*L of drug-free plasma at known concentrations. To construct a calibration curve, plasma samples containing matrine at 10, 50, 100, 250, 500, and 1000 ng/mL were analyzed. Low, medium, and high QC concentrations were 10, 100, and 1000 ng/mL, respectively. For the IS, 150 *μ*L of acetaminophen solution (1 *μ*g/mL in methanol) was added to the plasma samples, and the samples were vortex-mixed for 5 min followed by centrifugation at 12,000 rpm for 10 min. A 5 *μ*L aliquot of the supernatant was injected into the column for analysis.

### 2.4. HPLC-MS/MS Analysis

HPLC was performed on an Agilent 1100 HPLC system (Agilent Technologies, Inc., Santa Clara, CA, USA) and a reversed-phase column (Gemini C18, 50 mm × 4.60 mm, 5 *μ*m; Phenomenex, Torrance, CA, USA) was used with an isocratic mobile phase of 80% methanol and 20% distilled water at a flow rate of 0.15 mL/min. The column and autosampler were maintained at 25°C and 4°C, respectively. The total analytical run time was 5 min. Analyst software (v. 1.4.1; Applied Biosystems, Foster City, CA, USA) was used for the HPLC-MS/MS system control and analytical data processing.

### 2.5. Method Validation Procedure

The specificity of the method was determined using blank plasma samples obtained from six rats. The intraday and interday precision and accuracy were estimated by analyzing QC samples. The limits of precision and accuracy for acceptable data were within 15% for QC samples or within 20% for the lower limit of quantitation. Recovery was calculated by comparing the mean peak areas of matrine in plasma samples spiked before protein precipitation with the peak areas in samples spiked after protein precipitation. The matrix effect was determined by comparing the mean peak areas of matrine in plasma samples spiked before protein precipitation with the peak areas of analytes added directly to the mobile phase. Short-term, long-term, freeze–thaw, postpreparative, and stock stability tests were assessed at low (10 ng/mL) and high (1000 ng/mL) QC concentrations.

### 2.6. Animals

Sprague–Dawley (SD) rats (male, 7 weeks) were supplied by Daehan Biolink (Eumseong, Korea). The protocol of the present study was approved by the Ethics Committee of Animal Experimentation of Chungnam National University. SD rats (230–250 g) were housed under temperature-controlled conditions (21°C) with a 12 h light–dark cycle. The animals were given free access to food and water prior to experimentation, and a single oral dose of KIOM-MA128 was given after an overnight fast.

### 2.7. Application of Pharmacokinetic Study

KIOM-MA128 solutions were made using deionized water. Rats were given a single oral dose of KIOM-MA128 (4 g/kg; *n* = 5, 8 g/kg; *n* = 5). Blood samples (300 *μ*L) were collected by retroorbital bleeds at each time point 0.25, 0.5, 1, 2, 4, 8, 12, and 24 h after dosing. After collecting blood, each blood sample was centrifuged at 12,000 rpm for 10 min. Plasma was separated and stored at –70°C until analysis. To obtain pharmacokinetic parameters (*C*_max⁡_, *T*_max⁡_, AUC_inf⁡_, and *t*_1/2_), noncompartmental analysis was performed using Phoenix (Pharsight, Basel, Switzerland).

## 3. Results and Discussion

### 3.1. HPLC-MS/MS

**Figure 1:**
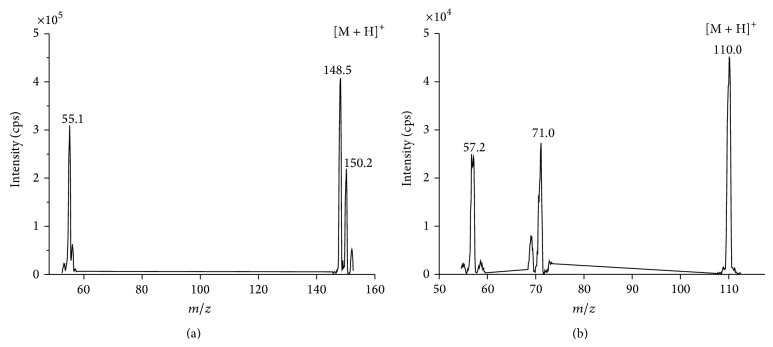
Tandem mass product ion spectra of matrine (a) and acetaminophen (b).

**Figure 2:**
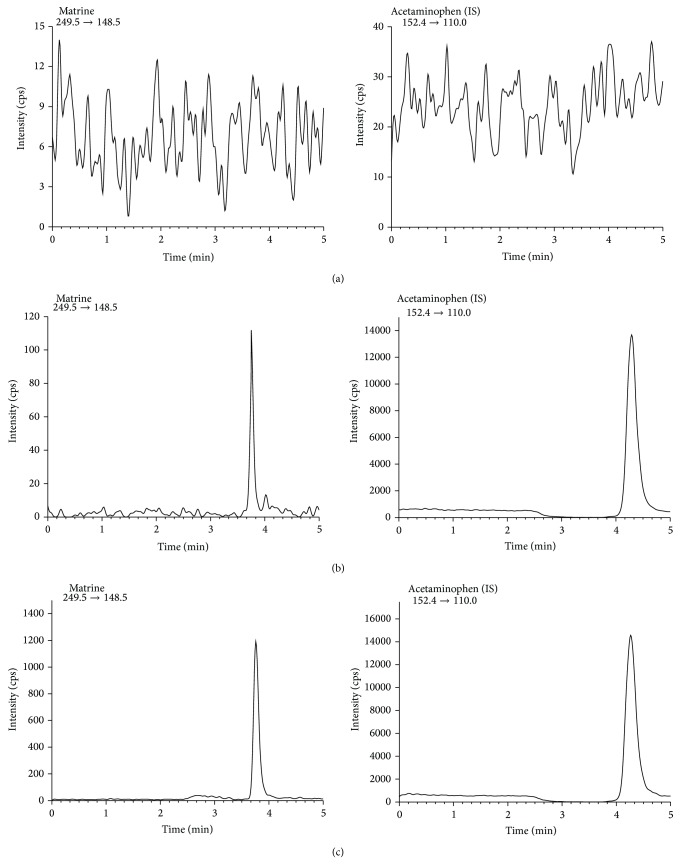
Chromatograms of rat blank plasma (a), rat plasma sample spiked with 10 ng/mL of matrine (LLOQ) (b), and a rat plasma sample obtained 2 hr after oral administration of KIOM-MA128 4 g/kg (c).

The precursor-product ion transitions used for matrine and IS were* m/z* 249.5→148.5 and 152.4→110, respectively (Figure 1). The retention times of matrine and the IS were 3.75 and 4.28 min, respectively. The chromatogram of a blank rat plasma sample analyzed by the described method is shown in Figure 2(a), where none of the matrix components interfered with the analytes under study or the IS at their corresponding retention times. The retention times of matrine and IS were reproducible throughout the study and no column deterioration was observed.

### 3.2. Method Validation

The calibration curve was linear from 10 to 1000 ng/mL for matrine. Linear regression analysis equation with a weighting of 1/*y* ^2^ was as follows: *y* = 0.000165*x* + 0.00039 (*r* ^2^ > 0.99). Intraday and interday precision and accuracy of QC samples are shown in Table 1. The accuracy (relative error; %RE) and precision (coefficients of variation; %CV) of matrine analysis ranged from 0.78% to 12.62% and from 7.22% to 13.69%, respectively. These results indicated that precision and accuracy of the present method were acceptable. The lower limit of quantitation was set at 10 ng/mL for matrine using 50 *μ*L of rat plasma; a representative chromatogram of the LLOQ is shown in Figure 2(b).

Matrix effects of matrine in low or high QC samples (*n* = 6) were 93.91 ± 1.58% and 100.02 ± 2.90%, respectively. Percent recoveries of matrine in low and high QC samples (*n* = 6) were 101.95 ± 3.35% and 105.94 ± 2.82%, respectively. Neither matrix effect nor the percent loss exceeded ±20%, and, therefore, no significant matrix effects or interference from endogenous compounds was caused by rat plasma.

### 3.3. Stability

The summary of stability under various conditions is presented in Table 2. The mean integrated peak areas of the low QC (10 ng/mL) and high QC (1000 ng/mL) samples were compared before and after the stability testing. There were no stability issues in the present method.

### 3.4. Application of PK

**Figure 3:**
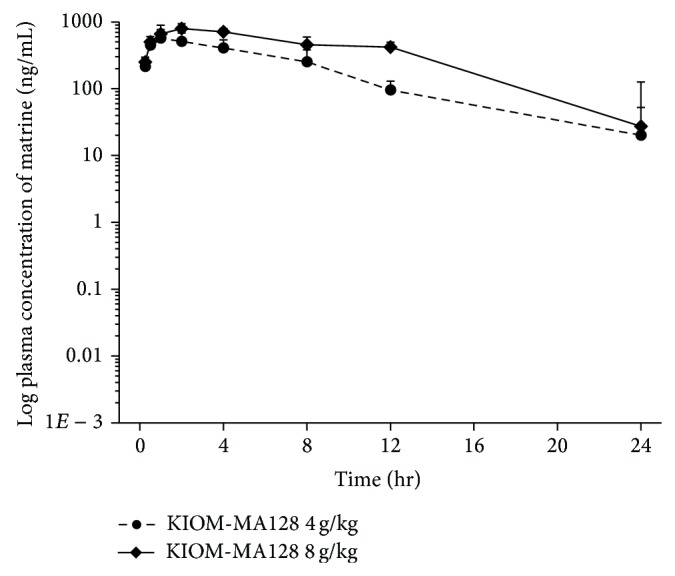
Time course of matrine log plasma concentration after oral administration of KIOM-MA128 4 g/kg (*n* = 5) and 8 g/kg (*n* = 5) in rats.

This method was successfully applied to analyze rat plasma in a pharmacokinetic study of KIOM-MA128. Chromatograms of the plasma samples obtained 2 h after oral administration of KIOM-MA128 (4 g/kg) in rats are shown in Figure 2(c). The pharmacokinetics of matrine in rats after oral administration of KIOM-MA128 (4 g/kg and 8 g/kg) is shown in Figure 3. The half-life (*t*_1/2_) calculated at the terminal phase and *T*_max⁡_ of matrine after administration of KIOM-MA128 were 4.29 ± 2.20 h and 1.8 ± 1.23 h, respectively. The maximum plasma concentrations (*C*_max⁡_) of matrine after administration of KIOM-MA128 at 4 g/kg and 8 g/kg were 595.10 ± 182.91 ng/mL and 850.46 ± 120 ng/mL, respectively. The area under the plasma concentration–time curve from 0 h to infinity (AUC_inf⁡_) after administration of KIOM-MA128 at 4 g/kg and 8 g/kg was 5336.77 ± 1503.84 ng/mL·h and 9583.10 ± 888.92 ng/mL·h, respectively.

## 4. Conclusions

The HPLC-MS/MS method described in this report is simple and rapid for determining the concentration of matrine after the administration of KIOM-MA128 in rats. All procedures for determining accuracy, precision, and stabilities of this method were in accordance with FDA regulations for the validation of bioanalytical methods. In this study, a method for determination of matrine which is one of the potential markers of KIOM-MA128 was developed and applied to an in vivo pharmacokinetic study in rats successfully. Herein, the first method for the determination of matrine after administration of KIOM-MA128 in rat plasma using HPLC-MS/MS has been described.

## Figures and Tables

**Table 1:** The intra- and interday precision and accuracy of matrine (*n* = 5).

Matrine	Nominal concentration (ng/mL)	Mean calculated concentration (ng/mL)	CV%	RE%
Intraday	10	10.08	13.69	0.78
	100	104.44	13.03	4.25
	1000	1117.23	9.68	10.49

Interday	10	10.25	7.22	7.11
	100	104.66	12.64	12.62
	1000	1054.33	9.99	9.99

**Table 2:** Stability test results of matrine in rat plasma.

Stability test	Storage condition	10 ng/mL	1000 ng/mL
		%stability	%stability
Short-term in plasma	Room temperature for 6 hr	108.36	90.69
Long-term in plasma	−70°C for 14 days	98.51	101.82
Freeze–thaw cycle in plasma	−70°C after the third cycle	92.06	90.86
Process (extracted sample)	4°C, for 24 hr	102.71	105.92
Stock solution	−20°C, for 14 days	98.51	107.33
